# Disease-Specific Comorbidity Clusters in COPD and Accelerated Aging

**DOI:** 10.3390/jcm8040511

**Published:** 2019-04-14

**Authors:** Filip J. J. Triest, Frits M. E. Franssen, Niki Reynaert, Swetlana Gaffron, Martijn A. Spruit, Daisy J. A. Janssen, Erica P. A. Rutten, Emiel F. M. Wouters, Lowie E. G. W. Vanfleteren

**Affiliations:** 1 CIRO+, Centre of Expertise for Chronic Organ Failure, 6085 NM Horn, The Netherlands; filiptriest@hotmail.com (F.J.J.T.); fritsfranssen@ciro-horn.nl (F.M.E.F.); martijnspruit@ciro-horn.nl (M.A.S.); daisyjanssen@ciro-horn.nl (D.J.A.J.); ericarutten07@gmail.com (E.P.A.R.); e.wouters@mumc.nl (E.F.M.W.); 2 Department of Respiratory Medicine, MUMC+, Maastricht University Medical Centre, 6229 ER Maastricht, The Netherlands; n.reynaert@maastrichtuniversity.nl; 3 Department of Respiratory Medicine, AZ Sint-Lucas, 9000 Ghent, Belgium; 4 NUTRIM School of Nutrition and Translational Research in Metabolism, Maastricht University Medical Centre+, 6229 ER Maastricht, The Netherlands; 5 Viscovery Software GmbH, 1130 Vienna, Austria; s.gaffron@viscovery.net

**Keywords:** comorbidity, multimorbidity, cluster, COPD, accelerated aging, telomere length

## Abstract

Background: Patients with chronic obstructive pulmonary disease (COPD) often suffer from multiple morbidities, which occur in clusters and are sometimes related to accelerated aging. This study aimed to assess the disease specificity of comorbidity clusters in COPD and their association with a biomarker of accelerated aging as a potential mechanistic factor. Methods: Body composition, metabolic, cardiovascular, musculoskeletal, and psychological morbidities were objectively evaluated in 208 COPD patients (age 62 ± 7 years, 58% males, FEV_1_ 50 ± 16% predicted) and 200 non-COPD controls (age 61 ± 7 years, 45% males). Based on their presence and severity, the morbidities were clustered to generate distinct clusters in COPD and controls. Telomere length in circulating leukocytes was compared across the clusters. Results: (co)morbidities were more prevalent in COPD patients compared to controls (3.9 ± 1.7 vs. 2.4 ± 1.5, *p* < 0.05). A “Psychologic” and “Cachectic” cluster were only present in the COPD population. “Less (co)morbidity”, “Cardiovascular”, and “Metabolic” clusters were also observed in controls, although with less complexity. Telomere length was reduced in COPD patients, but did not differ between the (co)morbidity clusters in both populations. Conclusions: Two COPD-specific comorbidity clusters, a “Cachectic” and “Psychologic” cluster, were identified and warrant further studies regarding their development. Accelerated aging was present across various multimorbidity clusters in COPD.

## 1. Introduction

Chronic obstructive pulmonary disease (COPD) is considered a complex, heterogeneous, and multicomponent condition [1]. Comorbidities are prevalent in COPD, impact on morbidity and mortality [2], and appear in various patterns [3]. Using a systematic and objective assessment, Vanfleteren et al. [4] identified five comorbidity clusters (a “Less Comorbidity”, “Cardiovascular”, “Metabolic”, “Psychologic”, and “Cachectic” cluster). Their identification suggested that differential pathophysiological pathways underlie these clusters. At a given genetic background, chronic diseases may develop and progress (at different speeds in various combinations) in response to common risk factors, such as smoking, alcohol, diet, pollution, and physical inactivity [5]. These environmental factors and intrinsic mechanisms may lead to accelerated aging [6]. Reduced telomere length, a surrogate marker for accelerated aging, was reported in circulating leukocytes in COPD [7], myocardial infarction, stroke, and diabetes [8]. While, some resemblance in comorbidity associations between COPD patients and (unmatched) controls was shown [9], the co-existence of multiple comorbidities in COPD patients and aging controls has not been explored by a unifying cluster analysis, nor by objective measurements. We hypothesized that comorbidity clustering in patients with COPD might differ from that of a non-COPD elderly population, and might relate to accelerated aging. We aimed to compare and validate the clustering of (co)morbidities in a sample of COPD and non-COPD elderly control subjects, and secondarily, to compare telomere length between the different (co)morbidity clusters in both COPD and non-COPD subjects.

## 2. Experimental Section

### 2.1. Study Design and Population

The current cross-sectional analysis is part of the “Individualized COPD Evaluation in relation to Ageing” (ICE Age) study [10] conducted between December 2010 and August 2016 in a tertiary care pulmonary rehabilitation center in the southeastern region of the Netherlands. Inclusion and exclusion criteria have been published before (Appendix A) [10]. In summary, stable COPD patients with moderate to severe airflow limitation and elderly controls without COPD or a history of debilitating chronic disease were included. All participants provided written informed consent prior to study participation. The study was conducted in accordance with the Declaration of Helsinki and good clinical practice guidelines. It was approved by the local ethics review board and registered on www.isrctn.com.

### 2.2. Assessments

Details of patient characterization, (co)morbidity assessment, and references of the (co)morbidity definitions are provided in Appendix A. Body composition, metabolic, cardiovascular, musculoskeletal, and psychological morbidities were objectively evaluated by the following measurements [4]: body mass index (BMI); fat-free mass index (FFMI by dual X-ray absorptiometry (DEXA); bone mineral density (BMD by DEXA) at the hip, lumbar spine, and whole body; pulse wave velocity (PWV); carotid intima-media thickness (c-IMT); peripheral systolic and diastolic blood pressure; fasting plasma glucose and serum insulin concentration to calculate the homeostasis model assessment method (HOMA) index; plasma creatinine concentration to calculate the glomerular filtration rate (GFR, using the simplified Modification-of-Diet-in-Renal-Disease equation); plasma triglyceride and high-density lipoprotein (HDL) levels; and Hospital Anxiety and Depression Scale (HADS). Telomere length in circulating leukocytes was determined by a monochrome multiplex quantitative PCR-procedure.

### 2.3. Definitions

Thirteen (co)morbidities were identified based on predefined cut-offs, mostly corresponding to our previous cluster analysis [4]. In summary: obesity was defined as BMI ≥ 30 kg/m^2^; hyperglycemia as plasma glucose concentration ≥ 5.6 mmol/L; insulin resistance as HOMA index ≥ 4.29/4.43 for women/men; dyslipidemia as plasma triglyceride concentration > 1.7 mmol/L or HDL < 1.29/1.03 mmol/L for women/men; hypertension as systolic blood pressure ≥ 140 mmHg or diastolic blood pressure ≥ 90 mmHg; arterial stiffness as PWV > 10 m/s; atherosclerosis as c-IMT > 0.9 mm; underweight as BMI < 21 kg/m^2^; low muscle mass as FFMI < 14.62/17.05 kg/m^2^ for women/men; osteoporosis as a T score < −2.5/−2.8 in women/men; renal impairment as estimated GFR < 60 mL/min/1.73 m^2^); and anxiety or depression as a HADS score ≥ 10 points. 

### 2.4. Statistical Analysis

Statistical analyses were performed using Viscovery SOMine 7.1 by Viscovery Software GmbH (www.viscovery.net; Vienna, Austria). Self-organizing maps (SOMs) were used to create an ordered representation for the presence of COPD and (co)morbidities. The SOM method can be viewed as a non-parametric regression technique that converts multidimensional data spaces into lower dimensional abstractions. It generates a non-linear representation of the data distribution and allows the user to identify homogenous data groups visually. Patients were primarily ordered by the presence/absence of COPD and secondarily by their overall similarity concerning their present (co)morbidities and also by the degree of its presence given by parameters from which the (co)morbidities were calculated. Based on the created SOM model, clusters were generated using the SOM–Ward Cluster algorithm that applies the classical hierarchical method of Ward on top of the SOM topology. Summary variables are presented as mean ± standard deviation for quantitative variables (except pack-years: median (interquartile range)), and percentage for discrete variables. Comorbidities, clinical characteristics, and telomere length were compared between: (1) The COPD and control group; (2) each (co)morbidity cluster and its corresponding group (COPD or control group); (3) each parallel (co)morbidity cluster in COPD and controls, using the integrated two-sided *t*-test with a confidence of 95%. Post-hoc in SPSS 19.0, additional binary logistic regression models were run within each group (unadjusted; adjusted for age; adjusted for age and sex), using the cluster as a dependent variable and telomere length as an independent variable.

## 3. Results

### 3.1. General Characteristics and (Co)Morbidities

**Figure 1:**
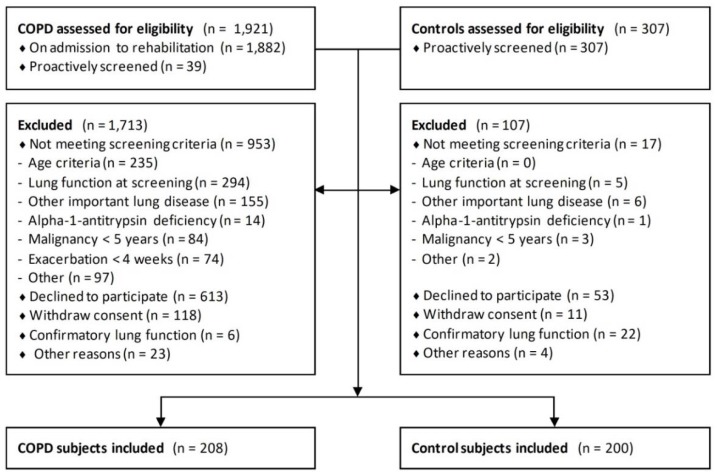
Subject enrollment. COPD = chronic obstructive pulmonary disease.

**Figure 2:**
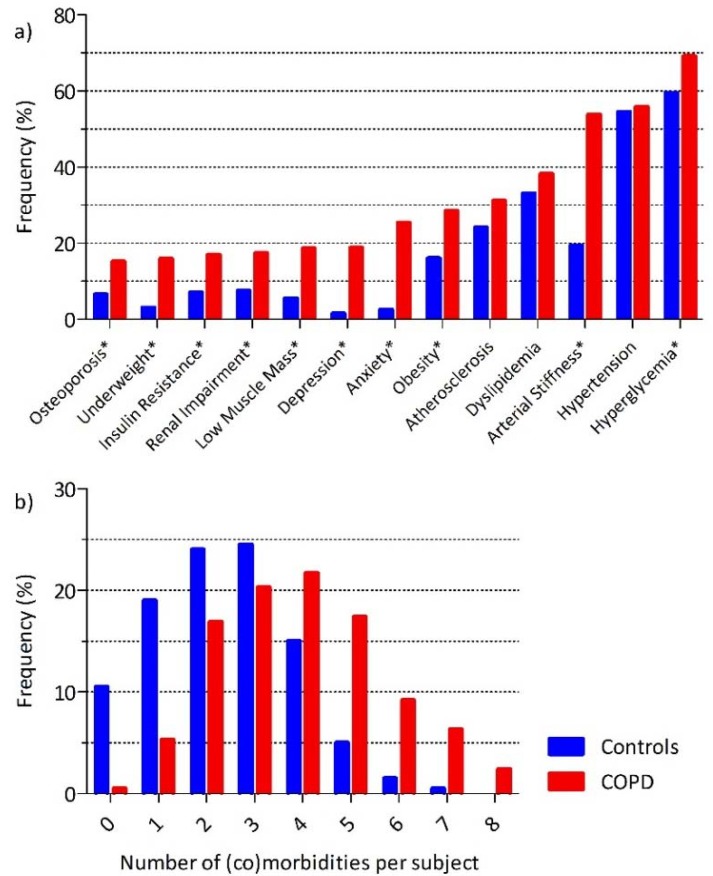
Frequencies of (co)morbidities. (**a**) Frequencies of (co)morbidities in COPD and controls: * More prevalent in the COPD group (*p* < 0.05); (**b**) Number of (co)morbidities per subject: mean number of (co)morbidities: 3.9 ± 1.7 in COPD vs. 2.4 ± 1.5 in controls (*p* < 0.05).

**Figure 3:**
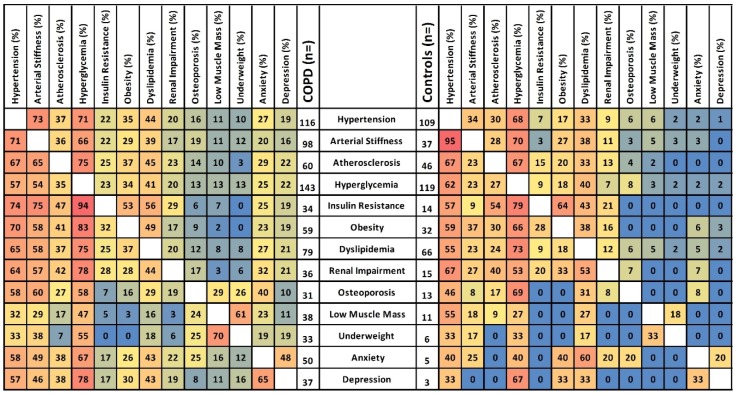
Co-existence of (co)morbidities in COPD and controls. The frequencies of co-existing (co)morbidities (%, ranging from 0 to 100%, horizontal row) in the presence of every index morbidity (vertical column) in COPD (left) and controls (right) are shown. These are descriptive data and do not offer a statistical comparison. For interpretation, the cells are color filled: red indicating the highest values, blue the lowest, and yellow for those in between.

Two-hundred-and-eight patients with COPD (62 ± 7 years) and 200 non-COPD controls (61 ± 7 years) were included (Figure 1). Patients with COPD were more often male and had a more extensive smoking history (Table 1). On average, both groups were slightly overweight, but controls had a lower cardiovascular risk. The COPD subjects had moderate to severe airflow limitation, moderately impaired diffusion capacity, increased static lung volumes, and impaired health status. Except for hypertension, atherosclerosis, and dyslipidemia, most (co)morbidities were more prevalent in patients with COPD (Figure 2a, baseline and missing data: Appendix A, Table A1). On average, patients with COPD had more (3.9 ± 1.7 versus 2.4 ± 1.5, *p* < 0.05, Figure 2b) and more overlapping (Figure 3) (co)morbidities than controls. 

### 3.2. (Co)Morbidity Clusters

**Figure 4:**
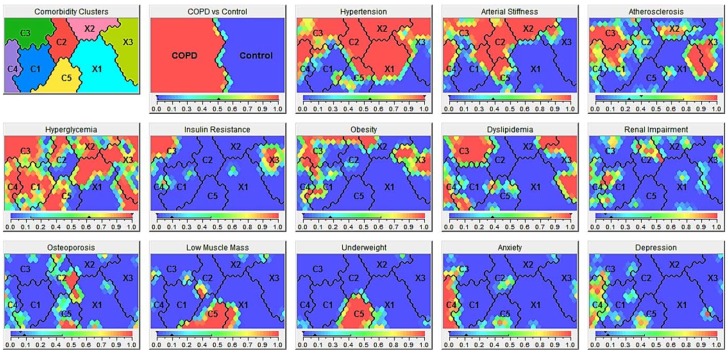
(Co)morbidity clusters in COPD and controls. Panels generated using Viscovery (Viscovery Software GmbH, Vienna, Austria). The Viscovery program primarily ordered all subjects on the map based on the presence or absence of COPD (second panel from the left in upper row) and secondarily by their overall similarity concerning the prevalent (co)morbidities and the degree (severity) of these (co)morbidities. The more subjects are comparable in terms of their (co)morbidity profile, the closer they are on the map. Contrarily, the more they differ, the further they are away from each other. Within the COPD patients, 5 clusters were identified (C1–C5), while there were only 3 in the controls (X1–X3). Within the panels showing the specific comorbidities, a red color indicates the presence of a specific comorbidity, while a blue color represents the absence of this condition. All panels represent the same map and a subject is always on the same place on the map. If the same node on the map colors red for “Low Muscle Mass” and “Underweight”, but blue for “Insulin Resistance”, a subject in that node, has a low muscle mass and is underweight, but does not have insulin resistance.

Five comorbidity clusters were identified in the COPD patients, and three (co)morbidity clusters in the non-COPD group (Figure 4).

#### 3.2.1. Comorbidity Clusters in COPD

Cluster C1 (“Less Comorbidity” cluster in COPD; *n* = 46 (22%)) had significantly fewer morbidities compared to the whole COPD group (Table 2). Cluster C2 (“Cardiovascular”, *n* = 27 (13%)) had the highest prevalence of hypertension, arterial stiffness, and osteoporosis, but a lower prevalence of metabolic features. Cluster C3 (“Metabolic”, *n* = 56 (27%)) had a higher prevalence of obesity, hyperglycemia, insulin resistance, and dyslipidemia, but also cardiovascular features. Cluster C4 (“Psychologic”, *n* = 40 (19%)) had the highest number of subjects with anxiety and depression. Cluster C5 (“Cachectic”, *n* = 39 (19%)) had the highest portion of subjects with underweight and low muscle mass. 

Functional exercise capacity, dyspnea scores, and frequency of exacerbations in the year prior to the study were not different among the comorbidity clusters (Table 3). However, patients in cluster C1 (“Less Comorbidity”) had less pack-years, less cardiovascular risk, and a better health-related quality of life. Cluster C2 (“Cardiovascular”) included older patients who were less frequently active smokers. Patients in cluster C3 (“Metabolic”) had a lower degree of airflow limitation, and static hyperinflation, and better diffusion capacity, while Framingham-defined cardiovascular risk was increased. Cluster 4 (“Psychologic”) had a higher proportion of women, a higher amount of pack-years, and a worse health status. Patients in cluster C5 (“Cachectic”) were more frequently active smokers, had worse pulmonary function with a higher degree of airflow limitation and static hyperinflation, but a lower Framingham-defined cardiovascular risk.

#### 3.2.2. (Co)Morbidity Clusters in Controls

Cluster X1 (“Less (co)morbidity” cluster in controls) was a large cluster (*n* = 97 of 200 non-COPD controls (49%)) and was characterized by a clearly fewer number of (co)morbidities (Table 4). In cluster X2 (“Cardiovascular”, *n* = 34 (17%)), hypertension and arterial stiffness was present in all subjects. Cluster X3 (“Metabolic”, *n* = 69 (35%)) had the highest portion of subjects with obesity, hyperglycemia, insulin resistance, and dyslipidemia. 

Cluster X1 (“Less (co)morbidity”) was a younger, predominantly female cluster with less pack-years, and a lower cardiovascular risk (Table 5). Cluster X2 (“Cardiovascular”) was older with a higher Framingham-defined cardiovascular risk. Cluster X3 (“Metabolic”) also had a high cardiovascular risk and accumulated more pack-years.

#### 3.2.3. Comparing (Co)Morbidity Clusters in COPD and Controls

Details of the comparison of the parallel (co)morbidity clusters “Less (Co)morbidity”, “Cardiovascular”, and “Metabolic” are shown in Table 4 and Table 5 (footnote ^2^ of the table). In summary, COPD subjects in the “Less (Co)morbidity” cluster (C1) had more (co)morbidities than those in the parallel control cluster (X1), including hyperglycemia, dyslipidemia, obesity, arterial stiffness, and renal impairment. The presence of (co)morbidities in the “Cardiovascular” clusters (C2, X2) was mainly comparable, except for a higher frequency of osteoporosis and less dyslipidemia in the COPD cluster C2. Both cluster C3 and X3 had a high prevalence of metabolic diseases. The “Metabolic” COPD cluster (C3) was older, more overweight, and had more (co)morbidities than cluster X3, in particular insulin resistance, hypertension, and arterial stiffness. 

### 3.3. Telomere Length

**Figure 5:**
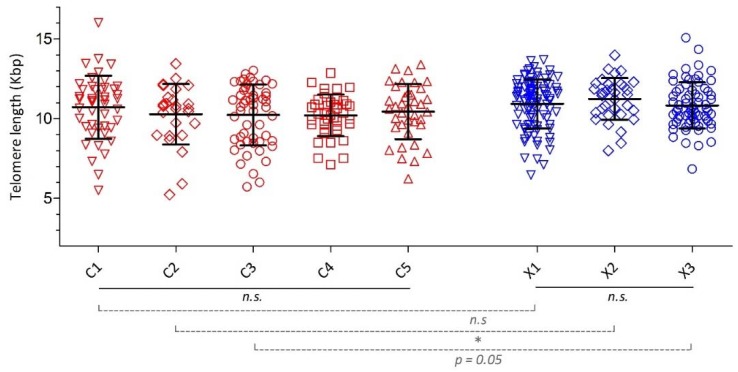
Telomere length across (co)morbidity clusters in COPD and controls. Scatter plots of telomere length across (co)morbidity clusters in COPD and controls, representing mean ± standard deviation. C1–5: COPD. X1–3: controls. n.s. = not significant. * Indicates significantly (*p* < 0.05) reduced telomere length in the COPD cluster compared to its parallel control cluster. For cluster C3 vs. X3: *p* = 0.05.

Telomere length was reduced in the patients with COPD compared to controls (Table 1). The mean telomere length of the (co)morbidity clusters did not differ significantly between the COPD clusters, nor between the non-COPD controls clusters (Figure 5 and Appendix A, Table A2), with similar results after adjusting for age and gender (Appendix A, Table A3 and Table A4). As for the parallel (co)morbidity clusters in COPD and controls, telomere length was comparable between both “Less (Co)morbidity” clusters C1 and X1, and significantly reduced in cluster C2 versus X2, while the difference failed to reach statistical significance for cluster C3 versus X3 (10.22 ± 1.90 versus 10.82 ± 1.45 kbp, *p* = 0.05) (Figure 5 and Appendix A, Table A2). 

## 4. Discussion

This study showed that a “Psychologic” and “Cachectic” comorbidity cluster were specifically related to COPD, and that “Cardiovascular”, “Metabolic”, and “Less-(co)morbidity” clusters were also present in aging controls, while it validates the previously identified comorbidity clusters [4]. Accelerated aging, measured by reduced telomere length, was present across various multimorbidity clusters in COPD, but was not limited to COPD-specific clusters.

Using a similar statistical approach, but a slightly different assessment of comorbidities, the current study identified a predominantly similar comorbidity clustering, compared to that in the CIROCO cohort [4], except for a few intriguing differences. Osteoporosis was now most prevalent in the “Cardiovascular” instead of the “Cachectic” cluster. This might be driven by the inclusion of arterial stiffness in the clustering model, which in turn relates to osteoporosis [11]. With respect to the clinical characterization, we confirmed the worse quality of life in the “Psychologic” cluster, the higher amount of smokers in the “Cachectic”, and former smokers in an older “Cardiovascular” cluster. In both studies, females tended to be more represented in the “Psychologic” or “Cachectic” cluster. 

A systematic review of studies that derived COPD phenotypes using unsupervised methods identified two recognizable phenotypes with poor longitudinal health outcomes across multiple studies [3]. A phenotype with moderate respiratory disease, obesity, cardiovascular, and metabolic comorbidities corresponds to the “Metabolic” cluster. Conversely, a phenotype with poor nutritional and health status, severe respiratory disease, and few cardiovascular comorbidities corresponds to the “Cachectic” cluster [3]. Indeed, underweight is prevalent in COPD, often co-occurs with low muscle mass [4], and is related to a more severe disease status [12]. Low body weight itself is also an independent risk factor for developing COPD [13].

The formation of emphysematous lesions might be promoted by cellular senescence which infers with tissue regeneration [14]. In turn, alveolar type II and endothelial cells from emphysematous lungs show shortened telomeres associated with increased cell senescence [15]. In a study in mice, it was reported that short telomeres lower the thresh-hold of cigarette smoke-induced damage, which implicated telomere length as a genetic susceptibility factor in emphysema [16]. On the other hand, emphysema severity is also associated with arterial stiffness [17] and shorter telomere length predict arterial stiffness [18]. On the other site of the phenotypic spectrum, telomere attrition has also been linked to type 2 diabetes [8]. These associations between telomere length and different comorbidities and emphysema, a widespread hallmark lesion in COPD [19], might explain the comparable presence of accelerated aging in the various comorbidity clusters in COPD. Also, in a subgroup of the BODE cohort, no differences in pulmonary function, nor BMI were found across telomere tertiles [20]. 

Anxiety and depression often co-occur [21,22] and are prevalent in COPD, especially in females [23]. Symptoms, poor health-related quality of life, behavioral factors, and social isolation in COPD contribute to the presence of mood disorders [24]. While the current study confirms the presence of an independent (predominantly female) “Psychologic” cluster [4], anxiety and depression are also associated to each other in non-COPD subjects [9]. The lower prevalence of mood disorders in the control population should be considered as a cause for the absence of a “Psychologic” cluster in the control population. 

Uniquely, this cluster study included elderly controls, allowing for a meaningful comparison of their—rigorously and objectively assessed—comorbidity profiles with those of patients with COPD, although the control population was, on average, one year younger. Most of them suffered from several morbidities, which underlines the importance of including a control population when studying multimorbidity from the perspective of an index disease [25]. Whereas three corresponding (co)morbidity profiles were identified in controls, these clusters were more complex in COPD. First, a higher number of comorbidities was seen in the “Less Comorbidity” COPD cluster, emphasizing the clinical need to consider comorbidities in all patients with COPD. Second, osteoporosis was more prevalent in the “Cardiovascular” COPD cluster, which could be linked to the use of oral corticosteroids in patients with COPD. Third, subjects in the “Metabolic” COPD cluster were older, more obese, and had more insulin resistance and cardiovascular comorbidity, which warrants future pathophysiologic research on cardiometabolic disease in COPD patients.

Considerations: Most COPD patients were recruited in a tertiary care pulmonary rehabilitation setting, which limits the external validity of the current findings. Reflecting clinical reality, patients with COPD had a more substantial smoking history than their controls. While influenced by smoking, reduced telomere length in COPD compared to smoking and non-smoking controls was consistently reported [7,20,26,27]. Telomere attrition is only one hallmark in the complex concept of aging [28], and the study was not powered to exclude differences in telomere attrition between the clusters. 

## 5. Conclusions

This study confirms previously identified clusters of objectively identified multiple comorbidities in a well-characterized cohort of patients with COPD and adds to this the comparison to a non-COPD elderly population, and the evaluation of telomere length between clusters, as a measure of biological aging. The current findings underline the complexity and heterogeneity of patients with COPD. Interestingly, exacerbation frequency, dyspnea, and functional exercise performance were similar amongst COPD comorbidity clusters, emphasizing again that detailed phenotyping is needed beyond the aforementioned clinical outcomes. In addition, a subgroup with low nutritional status and more severe lung function impairment, and a well-represented subgroup with psychological conditions are more specifically related to the presence of COPD and are important subgroups for targeted mechanistic and intervention studies. Reduced telomere length, is present across various comorbidity clusters in COPD, but did not seem a major determinant of (co)morbidity clustering itself.

## Figures and Tables

**Table 1:** Subject characteristics.

	COPD	Controls
Number of subjects, *n*	208	200
Male, *n* (%)	58	45 ^1^
Age, years	62 ± 7	61 ± 7 ^1^
BMI, kg/m^2^	26.9 ± 5.4	26.9 ± 3.4
Former smoker, %	65	55 ^1^
Current smoker, %	34	11 ^1^
Pack-years, *n*	43 (29–58)	6 (0–20) ^1^
Fram.10-yr. risk ≥ 30%, %	32	19 ^1^
FEV_1_, % predicted	50 ± 16	119 ± 15 ^1^
FEV_1_/FVC	41 ± 12	79 ± 5 ^1^
ITGV, % predicted	144 ± 33	100 ± 18 ^1^
KCO, % predicted	69 ± 22	101 ± 16 ^1^
MRC dyspnea grade	3.0 ± 1.0	NA
SGRQ, total score	55.0 ± 16.5	NA
6MWD, m	458 ± 131	NA
≥2 ex. previous year, *n* (%)	45	NA
Telomere length, kbp	10.37 ± 1.78	10.94 ± 1.47 ^1^

Abbreviations: BMI = body mass index; Fram.10-yr. risk = Framingham 10-year risk of overall cardiovascular disease; FEV_1_ = Forced expiratory volume in 1 s; FVC = Forced vital capacity. ITGV = Intrathoracic gas volume. KCO = Transfer factor for carbon monoxide per alveolar volume. MRC = Medical Research Council. SGRQ = St. George’s Respiratory Questionnaire. 6MWD = 6-min walking distance. ex. = Exacerbations. kbp = Kilobase pairs. NA = Not applicable. Summary variables are presented as mean ± standard deviation for quantitative variables, with exception of pack-years (median and interquartile range), and percentage for discrete variables. ^1^ *p* < 0.05 versus COPD group.

**Table 2:** Prevalence of comorbidities across the comorbidity clusters in COPD.

	C1	C2	C3	C4	C5
Cluster description	Less Comorbidity	Cardiovascular	Metabolic	Psychologic	Cachectic
Subjects, *n* (%)	46 (22%)	27 (13%)	56 (27%)	40 (19%)	39 (19%)
Comorbidities, *n*	2.5 ± 1.2 ^1^	3.4 ± 0.9	5.0 ± 1.3 ^1^	4.8 ± 1.9 ^1^	3.4 ± 1.4 ^1^
Hypertension, %	20 ^1^	96 ^1^	86 ^1^	55	28 ^1^
Arterial Stiffness, %	27 ^1^	92 ^1^	78 ^1^	53	29 ^1^
Atherosclerosis, %	33	16	47 ^1^	42	6 ^1^
Hyperglycemia, %	80	48 ^1^	82 ^1^	67	54 ^1^
Insulin resistance, %	5 ^1^	0 ^1^	52 ^1^	11	0 ^1^
Obesity, %	28	22	54 ^1^	25	0 ^1^
Dyslipidemia, %	26	0 ^1^	79 ^1^	44	15 ^1^
Renal Impairment, %	22	22	14	28 ^1^	3 ^1^
Osteoporosis, %	7	33 ^1^	7	15	24
Low muscle mass, %	0 ^1^	11	6 ^1^	3 ^1^	80 ^1^
Underweight, %	0 ^1^	0 ^1^	0 ^1^	5 ^1^	80 ^1^
Anxiety, %	2 ^1^	8 ^1^	9 ^1^	95 ^1^	14
Depression, %	9	0 ^1^	9 ^1^	59 ^1^	14

Summary variables are presented as mean ± standard deviation for quantitative variables and percentage for discrete variables. ^1^ *p* < 0.05 versus the COPD group.

**Table 3:** Clinical characteristics across the (co)morbidity clusters in COPD.

	C1	C2	C3	C4	C5
Cluster description	Less Comorbidity	Cardiovascular	Metabolic	Psychologic	Cachectic
Subjects, *n* (%)	46 (22%)	27 (13%)	56 (27%)	40 (19%)	39 (19%)
Male, %	63	67	64	43 ^1^	51
Age, years	61 ± 7	65 ± 7 ^1^	64 ± 6	61 ± 8	60 ± 6
BMI, kg/m^2^	27.2 ± 4.0	26.9 ± 3.5	31.1 ± 4.9 ^1^	27.5 ± 3.8	19.9 ± 2.0 ^1^
Waist circumference, cm	102 ± 12	102 ± 12 ^1^	112 ± 14	102 ± 15	82 ± 8 ^1^
Former smoker, %	59	89 ^1^	75	58	49 ^1^
Current smoker, %	39	11 ^1^	26	40	49 ^1^
Pack-years, *n*	39 (27–45) ^1^	40 (27–45)	45 (32–57)	58 (31–84) ^1^	43 (28–59)
Fram.10yr. risk ≥ 30%, %	18 ^1^	37	60 ^1^	26	10 ^1^
FEV_1_, % predicted	51 ± 16	49 ± 16	56 ± 13 ^1^	48 ± 15	40 ± 15 ^1^
FEV_1_/FVC	41 ± 12	40 ± 10	46 ± 11 ^1^	40 ± 11	36 ± 12 ^1^
ITGV, % predicted	139 ± 28	149 ± 30	128 ± 30 ^1^	144 ± 30	171 ± 32 ^1^
KCO, % predicted	67 ± 22	68 ± 17	80 ± 24 ^1^	66 ± 18	58 ± 18 ^1^
MRC dyspnea grade	3.0 ± 0.8	2.9 ± 1.1	2.9 ± 1.2	3.2 ± 0.9	3.0 ± 1.1
SGRQ, total score	48.3 ± 15.0 ^1^	56.1 ± 18.7	53.6 ± 15.1	66.2 ± 13.7 ^1^	52.2 ± 16.2
6MWD, m	445 ± 168	450 ± 159	462 ± 121	466 ± 100	465 ± 116
≥2 ex. previous year, %	46	33	44	48	53

Abbreviations: BMI = body mass index; Fram.10-yr. risk = Framingham 10-year risk of overall cardiovascular disease; FEV_1_ = Forced expiratory volume in 1 s; FVC = Forced vital capacity. ITGV = Intrathoracic gas volume. KCO = Transfer factor for carbon monoxide per alveolar volume. MRC = Medical Research Council. SGRQ = St. George’s Respiratory Questionnaire. 6MWD = 6-min walking distance. ex. = Exacerbations. kbp = Kilobase pairs. NA = Not applicable. Summary variables are presented as mean ± standard deviation for quantitative variables, with exception of pack-years (median and interquartile range), and percentage for discrete variables. ^1^ *p* < 0.05 versus COPD group.

**Table 4:** Prevalence of (co)morbidities across the (co)morbidity clusters in controls.

	X1	X2	X3
Cluster description	Less (Co)morbidity	Cardiovascular	Metabolic
Subjects, *n* (%)	97 (49%)	34 (17%)	69 (35%)
Comorbidities, *n*	1.4 ± 1.0 ^1,2^	3.8 ± 0.8 ^1^	3.1 ± 1.3 ^1,2^
Hypertension, %	44 ^1,2^	100 ^1^	46^2^
Arterial Stiffness, %	1 ^1,2^	100 ^1^	5 ^1,2^
Atherosclerosis, %	17 ^1,2^	28	32
Hyperglycemia, %	46 ^1,2^	68	74 ^1^
Insulin resistance %	1 ^1^	0	19 ^1,2^
Obesity, %	0 ^1,2^	21	36 ^1^
Dyslipidemia, %	0 ^1,2^	35 ^2^	78 ^1^
Renal Impairment, %	4 ^2^	12	10
Osteoporosis, %	9	6 ^2^	3
Low muscle mass %	7	6	3
Underweight, %	5	3	0
Anxiety, %	1	3	4
Depression, %	1 ^2^	0	3

Summary variables are presented as mean ± standard deviation for quantitative variables and percentage for discrete variables. ^1^ *p* < 0.05 versus the control group. ^2^ *p* < 0.05 versus the parallel COPD cluster.

**Table 5:** Clinical characteristics across the (co)morbidity clusters in controls.

	X1	X2	X3
Cluster description	Less (co)morbidity	Cardiovascular	Metabolic
Subjects, *n* (%)	97 (49%)	34 (17%)	69 (35%)
Comorbidities, *n*	1.4 ± 1.0 ^1,2^	3.8 ± 0.8 ^1^	3.1 ± 1.3 ^1,2^
Male, %	34 ^1,2^	59	54
Age, years	60 ± 6 ^1^	66 ± 6 ^1^	60 ± 6 ^2^
BMI, kg/m^2^	25.4 ± 2.4 ^1,2^	27.4 ± 3.8	28.7 ± 3.5 ^1,2^
Waist circumference, cm	89 ± 10 ^1,2^	95 ± 10 ^2^	98 ± 11 ^1,2^
Former smoker, %	53	62 ^2^	54 ^2^
Current smoker, %	8 ^2^	9	15
Pack-years, *n*	5 (0–16) ^1,2^	5 (0–22) ^2^	10 (0–25) ^1,2^
Fram.10yr. risk ≥30%, %	9 ^1^	33 ^1^	29 ^1,2^
FEV_1_, % predicted	119 ± 15 ^2^	122 ± 16 ^2^	118 ± 15 ^2^
FEV_1_/FVC	78 ± 5 ^2^	78 ± 5 ^2^	79 ± 5 ^2^
ITGV, % predicted	106 ± 20 ^1,2^	98 ± 17 ^2^	94 ± 14 ^1,2^
KCO, % predicted	99 ± 15 ^2^	100 ± 14 ^2^	105 ± 18 ^1,2^

Abbreviations: BMI = body mass index; Fram.10-yr. risk = Framingham 10-year risk of overall cardiovascular disease; FEV_1_ = Forced expiratory volume in 1 s; FVC = Forced vital capacity. ITGV = Intrathoracic gas volume. KCO = Transfer factor for carbon monoxide per alveolar volume. Summary variables are presented as mean ± standard deviation for quantitative variables, and percentage for discrete variables. ^1^ *p* < 0.05 versus the control group. ^2^ *p* < 0.05 versus the parallel COPD cluster.

## Appendix A

### Appendix A.1. Subject Enrollment

Inclusion and exclusion criteria have been published before [10]. COPD patients as well as non-COPD controls, 45–75 years of age were included. Following exclusion criteria were defined: any kind of carcinogenic pathology <5 years before study participation, other chronic lung diseases or previous lung surgery, alpha-1-antitrypsin deficiency, chronic use of oral corticosteroids >10 mg prednisolone/day, uncertainty about the willingness or ability of the subject to comply with the protocol requirements, and participation in a study involving investigational or marketed products concomitantly or <4 weeks prior to study entry. Inclusion criteria for patients with COPD were: moderate to severe airflow limitation (post-bronchodilator forced expiratory volume of 1s (FEV_1_) < 80% predicted, FEV_1_/forced vital capacity (FVC) < 70%) [2], and clinical stability, defined as absence of respiratory tract infection or exacerbation for <4 weeks before study entry. Control subjects should not have COPD or other diagnosis of debilitating chronic disease. Patients with COPD were recruited on admission to a tertiary care pulmonary rehabilitation center or from the same southeastern region of the Netherlands. Smoking and never-smoking (<1 pack-year) non-COPD controls were also recruited from this region.

### Appendix A.2. Assessments

#### Appendix A.2.1. General Characteristics

At study entry, subjects’ demographics, smoking status, and Framingham 10-year risk for cardiovascular disease [29] were documented. In subjects with COPD, the number of exacerbations in the past year, dyspnea (Medical Research Council (MRC) grade) [30]), six-minute walk distance [31], and health related quality of life (St. George’s Respiratory Questionnaire (SGRQ) [32]) were also assessed. Baseline post-bronchodilator spirometry using a standardized spirometer, measurement of static lung volumes, and carbon monoxide transfer factor lung function (Masterlab^®^, Jaeger, Würzburg, Germany) was performed in all subjects.

#### Appendix A.2.2. Assessments of (Co)Morbidities

The following clinically relevant variables were assessed:

- Body mass index (BMI = body weight in kg/(height in m)^2^. Height was measured to the nearest 0.1 cm. Body weight was assessed to the nearest 0.1 kg after emptying the bladder and with the subjects standing barefoot and wearing light indoor clothing.
- Fat-free mass index (FFMI) = fat free mass in kg (=lean mass + bone mineral content by dual X-ray absorptiometry (DEXA) scan (Lunar Prodigy, GE Healthcare)) divided by (height in m)^2^ [33].
- Bone mineral density (BMD by DEXA scan) at the hip, lumbar spine, and whole body [34].
- Anxiety and depression were assessed by using the Hospital Anxiety and Depression Scale (HADS), a validated and reliable measurement instrument used widely in medically ill patients to screen for clinically relevant symptoms of anxiety and/or depression [35]. The HADS is a self-administered questionnaire, consisting of 14 questions. The HADS is divided into an anxiety subscale (HADS-A) and a depression subscale (HADS-D), both containing seven items. Total scores for each subscale can range from 0 (optimal) to 21 (worst) points.
- Pulse wave velocity (PWV) assessment methods were similar in the CIROCO and ICE Age studies and have been published before [36]. Radial artery waveforms were recorded with a high-fidelity micromanometer (Millar Instruments, Houston, TX, USA). The APWV was measured by recording ECG-gated carotid and femoral artery waveforms. The Sphygmocor systems software helped to assure the quality of the pulse wave measurement. A detailed screen showed 10 s of recorded and analyzed waveforms which can be examined to assess overall consistency of the waveforms. In addition, a detailed report helped to interpret the consistency of the waveforms during the 10-s measurement. Only when the pulse height and diastolic variation was less than 5%, the average pulse height was more than 100 units, the augmentation index was less than 50%, T1 was between 80 and 150 ms, and the overall “quality index” above 80 points, data were retained. After marking the exact location measurements, it was repeated three times for securing reproducibility. A measurement was accepted when it was reproducible three times with minimal variation as judged by the biomedical technologist. The retained APWV was the mean of the three measurements. Shortest distances from manubrium to the marked location on the femoral artery (via the navel) were measured. Wave transit time was calculated by the system software, using the R-wave of the simultaneously recording electrocardiography as reference frame. The APWV was determined by dividing the distance between the two recording sites by the wave transit time.
- Carotid intima-media thickness (c-IMT) was assessed using high-resolution B-mode ultrasound with a 10-MHz linear transducer (Art. LabEsoatePicus, Pie-medical Netherlands/Italy). The ultrasound device was connected to an acquisition modem with an automatic boundary detection system (Art.LabExoatePicus). Carotid IMT was thus quantified semi-automatically, reducing the interobserver variability [37]. With the patient in a supine position, measurements of the carotid IMT were performed throughout 10-mm segments across the bifurcation free of visual plaques. The probe was moved to obtain measurements of the left common carotid artery at 4 angles (180°, 150°, 120°, and 90°). For each measured segment, mean and maximum IMT values were acquired automatically throughout the 10-mm vessel length. The average of segmental maximum carotid values was determined as carotid IMT per patient.
- Peripheral systolic and diastolic blood pressure measurements were performed early in the morning after 15 min in a resting supine position. A small head pillow was accepted. Peripheral blood pressure was measured three times with intervals of 5 min.
- Laboratory analysis: A venous blood sample was collected in the fasted state. Serum, plasma, and peripheral blood mononuclear samples were stored at −80 °C prior to their analysis. Glucose, creatinine, high-density lipoprotein (HDL), and triglycerides were determined in all subjects. Fasting plasma glucose and serum insulin concentration were used to calculate the homeostasis model assessment method (HOMA index: fasting serum insulin (μU/mL) × fasting plasma glucose (mmol/L)/22.5), plasma creatinine concentration to calculate the estimated glomerular filtration rate (eGFR) using the simplified Modification of Diet in Renal Disease (MDRD) equation [38].

#### Appendix A.2.3. Telomere Length

Telomere length of blood leucocytes was determined by a monochrome multiplex quantitative PCR procedure as described by Cawthon [39] with DNA of two reference cell lines with known telomere length [40] included in each run. Primers used for the telomeres were: Telg (ACACTAAGGTTTGGGTTTGGGTTTGGGTTTGGGTTAGTGT), and Telc (TGTTAGGTATCCCTATCCCTATCCCTATCCCTATCCCTAACA). For the single copy reference gene beta-globin the primers used were: hbgu (CGGCGGCGGGCGGCGCGGGCTGGGCGGcttcatccacgttcaccttg), and hbgd (GCCCGGCCCGCCGCGCCCGTCCCGCCGgaggagaagtctgccgtt).

### Appendix A.3. Definitions

- COPD and grades of severity were classified according to the GOLD document [41].
- OBESITY was defined as a BMI equal to or above 30 kg/m^2^ according to the WHO international classification of BMI [42].
- HYPERGLYCEMIA was defined as fasting glucose level equal to or above 5.6 mmol/L according to the American Diabetes Association [43].
- INSULIN RESISTANCE. The homeostasis model assessment method (HOMA 1 IR index) is a simple and validated method to assess insulin sensitivity [44]. It is also an independent predictor of cardiovascular disease in patients with type 2 diabetes mellitus [45]. It was calculated as follows: HOMA index = fasting serum insulin (μU/mL) × fasting plasma glucose (mmol/L)/22.5. Cut-off values depended on geographic variation and gender, and defining a local population, percentile-based cut-off is recommended for the HOMA index and/or hyperinsulinemia [46,47]. Therefore, we used the 75th percentile of the HOMA 1 IR of the Hoorn study, a large Dutch cohort study in men and women aged 50 to 75 years was used to define insulin resistance [48]. We based the cut-off on the entire population of the Hoorn study without exclusion of patients with diabetes mellitus and/or prevalent cardiovascular disease and used separate values for males and females. These data were provided by personal communication with JM Dekker. Insulin resistance was defined as a HOMA 1 IR equal to or above 4.43 for males and 4.29 for females.
- DYSLIPIDEMIA was defined as a triglyceride level above 1.7 mmol/L or a HDL cholesterol level below 1.03 mmol/L in males or below 1.29 mmol/L in females [49].
- HYPERTENSION was defined as a systolic blood pressure equal to or above 140 mmHg or diastolic pressure equal to or above 90 mmHg, in line with the European Society of Hypertension (ESH)/European Society of Cardiology (ESC) guidelines [50].
- ARTERIAL STIFFNESS was defined as pulse wave velocity (PWV) > 10 m/s, as proposed by the expert consensus document of the Artery Society, the European Society of Hypertension Working Group on Vascular Structure and Function, and the European Network for Noninvasive Investigation of Large Arteries [51]. This value is at the upper part of the second quartile in the Framingham Heart Study and represents in this general population with a mean age of 63 years about 4% risk for a first major cardiovascular event within the next 8 years [52].
- ATHEROSCLEROSIS was defined as a maximal carotid intima media thickness (IMT) > 0.9 mm, which is according to the ESH/ESC guidelines compatible with subclinical organ damage [50].
- UNDERWEIGHT was defined as a BMI lower than 21 kg/m^2^. The purpose of a BMI cut-off point is to identify within a population, the proportion of people with a high risk of an undesirable health state. Hence, a BMI lower than 21 kg/m^2^, which has shown to be independently associated with one-year mortality in COPD, is an appropriate cut-off [42,53].
- LOW MUSCLE MASS was defined as a fat free mass index (FFMI) less than 17.05 kg/m^2^ for men or less than 14.62 kg/m^2^ for women, according to the lowest 10th percentile of the general population as reported in the Copenhagen City Heart Study [54], which is also supported by the Nutritional assessment and therapy in COPD statement of the European Respiratory Society statement [33].
- OSTEOPOROSIS was assessed by DEXA scanning and established if the lowest T score of the lumbar spine or hip was lower than −2.5 for females and lower than −2.8 for males, according to Dutch guidelines [55].
- RENAL IMPAIRMENT was defined as an eGFR less than 60 mL/min/1.73 m^2^, which was estimated, using the simplified Modification of Diet in Renal Disease (MDRD) equation [38], corresponding with stage 3 chronic kidney disease according to the National Kidney Foundation Kidney Disease Outcome Quality Initiative (NKF KDOQI) guidelines [56].
- ANXIETY was assessed, using the Hospital Anxiety and Depression Scale (HADS). A score equal to or greater than 10 points on the items related to anxiety, was considered to indicate symptoms of anxiety [35].
- DEPRESSION was also assessed, using the HADS. A score equal to or greater than 10 points on the items related to depression, was considered to indicate symptoms of depression [35].

### Appendix A.4. Baseline Data

**Table A1:** Comparison of baseline data between COPD and controls.

	COPD	All Controls
Plasma glucose level, mmol/L	6.2 ± 1.6	5.9 ± 0.9 ^1^
Systolic blood pressure, mmHg	145 ± 22	143 ± 20
Diastolic blood pressure, mmHg	84 ± 9	84 ± 10
Pulse wave velocity, m/s	10.6 ± 2.7	8.8 ± 2.2 ^1^
Plasma triglyceride level, mmol/L	1.5 ± 0.8	1.4 ± 1.0
Plasma HDL level, mmol/L	1.6 ± 0.5	1.6 ± 0.5
cIMT, µm	836 ± 181	800 ± 156 ^1^
HOMA index	3.4 ± 6.0	2.0 ± 1.4 ^1^
eGFR, mL/min/1.73 m^2^	73.6 ± 15.8	77.6 ± 13.6 ^1^
BMI, kg/m^2^	26.9 ± 5.4	26.9 ± 3.4
FFMI, kg/m^2^	17.6 ± 2.6	18.1 ± 3.1
Lowest T score	−1.7 ± 1.0	−0.9 ± 1.1 ^1^
HADS anxiety, score	6.6 ± 4.2	3.5 ± 2.7 ^1^
HADS depression, score	6.0 ± 3.9	2.0 ± 2.2 ^1^

Abbreviations: HDL = high density lipoprotein; cIMT= carotid intima media thickness; HOMA = homeostasis model assessment method; GFR = glomerular filtration rate; BMI = body mass index; FFMI = fat-free mass index; HADS = Hospital Anxiety and Depression Scale. Summary variables are presented as mean ± standard deviation for quantitative variables, and percentage for discrete variables. ^1^ *p* < 0.05 versus COPD group. Missing data for subjects with COPD: 26 subjects with COPD lacked an acceptable quality pulse wave velocity measurement; 15 subjects lacked an acceptable quality carotid intima-media thickness measurement, 11 did not fill out the entire hospital anxiety and depression scale, 1 subject did not give a blood sample, for 6 additional patients insulin levels could not be measured, and 4 subjects did not have a dual-energy X-ray absorptiometry. Missing data for controls: 9 control subjects lacked an acceptable quality pulse wave velocity measurement; 8 subjects lacked an acceptable quality carotid intima-media thickness measurement; insulin level could not be measured for one subject; 1 did not fill out the entire hospital anxiety and depression scale.

### Appendix A.5. Telomere Length

**Table A2:** Telomere length across the (co)morbidity clusters in COPD and controls.

	C1	C2	C3	C4	C5	X1	X2	X3
Subjects, *n* (%)	46 (22%)	27 (13%)	56 (27%)	40 (19%)	39 (19%)	97 (49%)	34 (17%)	69 (35%)
Telomere length, kbp	10.71 ± 1.99	10.27 ± 1.90	10.22 ± 1.90	10.20 ± 1.31	10.44 ± 1.73	10.92 ± 1.54	11.23 ± 1.32 ^1^	10.82 ± 1.45 ^2^

Abbreviations: C = COPD cluster. X = control cluster. kbp = kilobase pairs. Summary variables are presented as mean ± standard deviation for quantitative variables and percentage for discrete variables. The mean telomere length of the (co)morbidity clusters did not differ significantly between the COPD clusters, nor between the non-COPD controls clusters. ^1^ *p* < 0.05 versus the parallel COPD cluster. ^2^ *p* = 0.05 versus the parallel COPD cluster.

**Table A3:** Telomere length and morbidity clusters in COPD: binary logistic regression.

	Crude OR	Age Adj. OR	Age–Sex Adj. OR
C1	1.15 (0.95–1.40)	1.15 (0.94–1.39)	1.15 (0.95–1.40)
C2	0.96 (0..77–1.21)	0.99 (0.78–1.25)	0.99 (0.78–1.26)
C3	0.94 (0.79–1.12)	0.95 (0.80–1.13)	0.95 (0.80–1.14)
C4	0.94 (0.77–1.14)	0.93 (0.76–1.12)	0.91 (0.75–1.11)
C5	1.03 (0.84–1.25)	1.01 (0.83–1.23)	1.01 (0.82–1.23)

Odds ratios (ORs) from binary logistic regression models run within the COPD group, using the cluster as a dependent variable and telomere length as an independent variable: 1. unadjusted; 2. adjusted for age; 3. adjusted for age and sex. Odds ratios (95% confidence interval) are reported.

**Table A4:** Telomere length and morbidity clusters in controls: binary logistic regression.

	Crude OR	Age Adj. OR	Age–Sex Adj. OR
X1	0.98 (0.81–1.19)	0.99 (0.82–1.20)	1.01 (0.83–1.23)
X2	1.19 (0.91–1.54)	1.22 (0.91–1.63)	1.21 (0.91–1.61)
X3	0.92 (0.75–1.12)	0.92 (0.76–1.13)	0.91 (0.74–1.11)

Odds ratios (ORs) from binary logistic regression models performed within the control group, using the cluster as a dependent variable and telomere length as an independent variable: 1. unadjusted; 2. adjusted for age; 3. adjusted for age and sex. Odds ratios (95% confidence interval) are reported.
